# Association and Haplotype Analyses of Positional Candidate Genes in Five Genomic Regions Linked to Scrotal Hernia in Commercial Pig Lines

**DOI:** 10.1371/journal.pone.0004837

**Published:** 2009-03-16

**Authors:** Zhi-Qiang Du, Xia Zhao, Natascha Vukasinovic, Fernanda Rodriguez, Archie C. Clutter, Max F. Rothschild

**Affiliations:** 1 Department of Animal Science and Center for Integrated Animal Genomics, Iowa State University, Ames, Iowa, United States of America; 2 Newsham Choice Genetics, West Des Moines, Iowa, United States of America; 3 Monsanto Company, Saint Louis, Missouri, United States of America; University Medical Center Groningen, Netherlands

## Abstract

Scrotal hernia in pigs is a complex trait likely affected by genetic and environmental factors. A large-scale association analysis of positional and functional candidate genes was conducted in four previously identified genomic regions linked to hernia susceptibility on *Sus scrofa* chromosomes 2 and 12, as well as the fifth region around 67 cM on chromosome 2, respectively. In total, 151 out of 416 SNPs discovered were genotyped successfully. Using a family-based analysis we found that four regions surrounding *ELF5*, *KIF18A*, *COL23A1* on chromosome 2, and *NPTX1* on chromosome 12, respectively, may contain the genetic variants important for the development of the scrotal hernia in pigs. These findings were replicated in another case-control dataset. The SNPs around the *ELF5* region were in high linkage disequilibrium with each other, and a haplotype containing SNPs from *ELF5* and *CAT* was highly significantly associated with hernia development. Extensive re-sequencing work focused on the *KIF18A* gene did not detect any further SNPs with extensive association signals. These genes may be involved in the estrogen receptor signaling pathway (*KIF18A* and *NPTX1*), the epithelial-mesenchymal transition (*ELF5*) and the collagen metabolism pathway (*COL23A1*), which are associated with the important molecular characteristics of hernia pathophysiology. Further investigation on the molecular mechanisms of these genes may provide more molecular clues on hernia development in pigs.

## Introduction

Most of the inguinal and scrotal hernias (*hernia inguinalis* and *scrotalis*) are birth defects in human, under the control of genetic and environmental factors. However, the molecular mechanism of etiology remains elusive [1]–[5]. In the pig breeding industry, infrequent incidence of inguinal and scrotal hernias can happen for certain pig breeds and lines and genetic factors are believed to drive the hernia development [6]–[8]. Without clinical examination, these two defects cannot be easily distinguished, and most of the time, this is the case.

Genome-wide association analyses (GWA) have now been frequently applied in the search for human disease susceptibility loci [9]. This approach could not be realized without the advancement of next-generation sequencing and genotyping technologies, as well as the massive efforts spent in the annotation of the structural and functional properties of the human genome (HapMap and ENCODE projects) [10], [11]. Moreover, attempts to identify the causal genes or gene subnetworks for a disease-linked genomic region have employed large-scale candidate gene association analyses [12]–[15], linkage disequilibrium (LD) fine mapping [16], and more recently, reversely engineered molecular network methods [17].

Our previous genome-wide linkage studies have identified several genomic regions related to the hernia incidence in Pietrain-based lines [18], [19]. The quantitative trait loci (QTL) regions on *Sus scrofa* chromosomes (SSC) 2 and 12 have been replicated in seven other independent paternal families derived from three commercial Pietrain-based pig lines [19]. Moreover, in the same genomic regions on SSC2 and SSC12, QTL have been identified in another pig breed, Landrace, using affected sib pairs, which suggests that common genetic origins may be involved even for different pig populations [20]. We continued to refine the interesting regions by LD analysis, and found three independent regions, at approximately 3, 42 and 65 cM on SSC2, and the first 20 cM region on SSC12, with genes segregating for the risk to develop inguinal and scrotal hernias [19].

Since the pig whole genome sequencing project is not yet completed, we had to use all available sequence information in pigs, as well as the comparative information from the human genome. Taking into consideration the practical concerns of experimental power in the design of GWA and candidate gene association analyses in human disease studies [9], [21], [22], and the genomic distribution of LD status in pigs [23], we selected 99 positional candidate genes located in the aforementioned interesting regions on SSC2 and SSC12 to conduct a regional large-scale resequencing and association study for the genetic causes of scrotal/inguinal hernia. The possible dysfunction of these genes can result in the aberrant collagen metabolism (the most probable reason considered for hernia development) [24]–[27], the smooth muscle breakdown [28], [29], an altered apoptosis pathway [30], the sex hormone deregulation [31]–[33], and the dedifferentiation of fibroblasts derived from the stem cells during epithelial-mesenchymal transition (EMT) [34].

## Results

**Figure 1:**
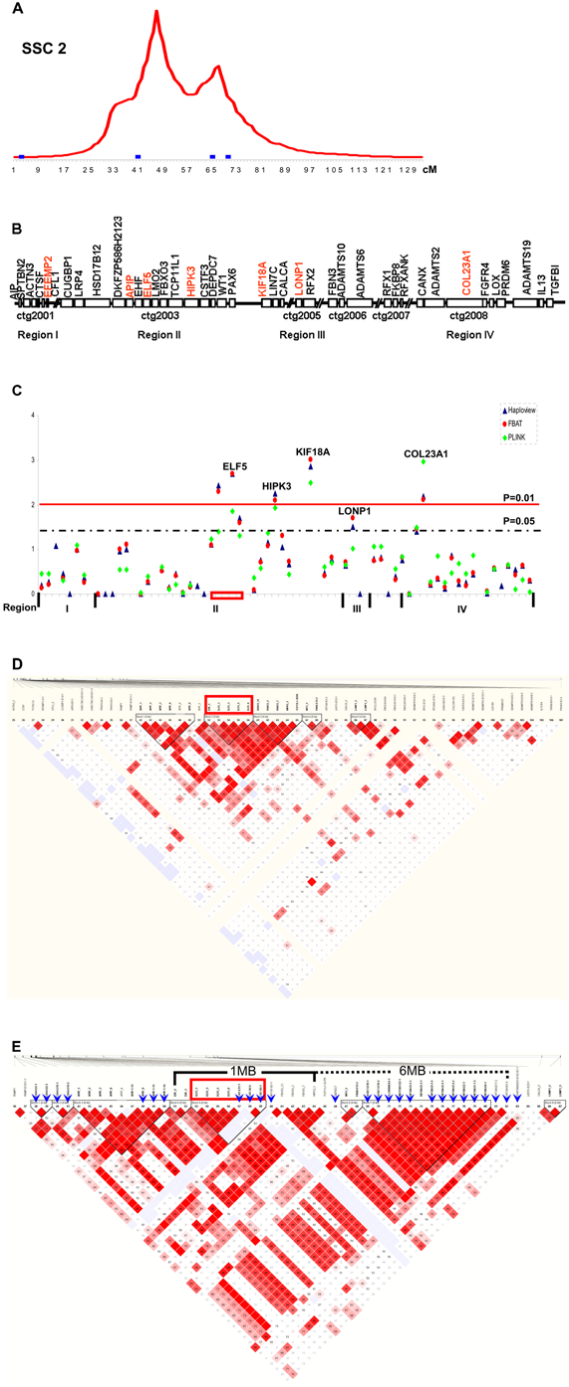
Extensive positional candidate gene analyses of four porcine genomic regions for hernia development on SSC2. A, Hernia QTL on SSC2. Genomic region around 48 cM was significantly linked to risk of hernia development. Three regions (I, at 3 cM; II at 42 cM; III, at 66 cM) and a fourth region (IV at 70 cM) could be potentially linked to scrotal hernia. These regions were indicated by the blue frames. B, The gene map was built based on the latest porcine physical FPC (fingerprint contigs) map (Sanger Porcine Genome Sequencing Project) (Table S1). Genes in contigs 1, 3, 5 and 8 on SSC2 were selected to cover the four regions, respectively. Contigs 6 and 7 were also used to search for candidate genes. C, Single marker association analyses. SNPs of genes located in region II were significantly associated with pig hernia in Pietrain-based line. The small red box includes SNPs in ELF5 significantly associated with scrotal hernia. D, LD heatmap was constructed by Haploview. Genes in region II were found to be in high LD with each other. The red box indicates the common haplotype formed by ELF5-1, ELF5-3, ELF5-5, ELF5-8 and EHF1 was significantly associated with scrotal hernia. E, 26 new SNP markers were added into the region II region (blue arrowhead), with focus on the 1 MB region including *EHF-ELF5-CAT*, and the 6 MB region covering *KIF18A*. Re-sequencing work on *KIF18A* did not detect any further association signal. The red box defines the haplotype composed of ELF5-1, ELF5-3, ELF5-5, ELF5-8, CAT-E11-1, CAT-5U-5 and CAT-5U-1 to be highly significantly associated with scrotal hernia.

Based on the nucleotide sequence information provided by the markers in the three regions associated with scrotal hernia on SSC2 at approximately 3, 42 and 65 cM, which existed in genomic DNA sequences (NCBI accession numbers: BH021488, DQ648562 and CL352219), we blasted them against the NCBI nucleotide database, and selected a first set of four important candidate genes distributed in the three specific sub-regions, i.e. the homeodomain interacting protein kinase 3 (*HIPK3*) and the complement regulatory protein CD59 molecule (*CD59*) in region II, the cathepsin F (*CTSF*) in region I, and the mitochondrial lon peptidase 1 (*LONP1*) (Figure S1). Then new candidate genes were selected on both ends of the first set of genes, combining the comparative genomic information between human and pig (Table S1), and the information from the constructed gene networks, too (Figure S2). This further enabled us to select additional positional candidate genes from HSA5q35 potentially involved in human inguinal hernia [35], e.g. the collagen type XXIII, alpha 1 (*COL23A1*) and the ADAM metallopeptidase with thrombospondin type 1 motif, 2 (*ADAMTS2*), which likely map to the region surrounding 67 cM on SSC2 potentially linked to hernia susceptibility (Figure 1).

In order to give a better coverage over the interesting regions, we have chosen 99 genes in total, both functionally and positionally, from the five interesting regions linked with scrotal hernia in pigs on SSC2 and SSC12. For these 99 genes, we discovered 451 SNPs (dbSNP accession numbers: 107794144–107794595) (average: 4.21 SNPs per gene, range: 1–27) (278 SNPs in 64 genes on SSC2, 138 SNPs in 35 genes on SSC12, respectively). Due to the cost and development of multiplexes, we have assayed 181 SNPs from 89 genes in six iPLEXes by the Sequenom platform (average: 30 SNPs per iPLEX, range: 28–37). After quality evaluation of the genotype data, 151 SNPs (dbSNP accession numbers: 107794444–107794595) are in Hardy-Weinberg equilibrium (p>0.01), with acceptable genotyping success rates (>85%), and no deviation of Mendelian inheritance existed in our pig families for these SNPs. We adopted the iterative strategy for the practical genotyping procedure. The genotype data were analyzed after one round of genotyping, and then based on the results we got, we chose new SNPs for new assays, according to their positions and functional importance, as well as the requirement of maximizing the number of genes included in the iPLEXes to be studied to increase the marker coverage density.

In the family-based Pietrain lines, we found several SNPs in the four regions on SSC2 were significantly associated with scrotal hernia incidence, respectively, after single marker association analysis (Figure 1, Table 1, Table S2). One SNP in the epidermal growth factor-containing fibulin-like extracellular matrix protein 2 (*EFEMP2*) located in region I (Figure 1C), one SNP in *LONP1* in region III, and one SNP in *COL23A1* around 67 cM were found to be significantly associated with scrotal hernia, respectively (Table 1, Table S2, Figure 1C). Interestingly, four SNPs, ELF5-1, ELF5-8, HIPK3-1, and KIF18A-E3-3, in three genes located in or close to region II, the E74-like factor 5 (*ELF5*), *HIPK3* and the kinesin family member 18A (*KIF18A*), were highly significantly associated with scrotal hernia (p<0.01) (Table 1, Table S2). Even after FDR correction, they were still significant at p<0.05. The localization of the four SNPs were right under the hernia QTL region around 42 cM on SSC2 (Figure 1A), and they were in high LD with each other (Figure 1D). Furthermore, a common haplotype formed by five SNPs, ELF5-1, ELF5-3, ELF5-5, ELF5-8 and EHF1, from *ELF5* and a neighboring gene EHF, but not *HIPK3* and *KIF18A*, was significantly associated with scrotal hernia (p<0.001).

**Table 1:** Association analysis results on two datasets: the family-based data and the case-control data.

Chr.	Gene	SNP	Family-based analysis			Case-control		
			FBAT	Haploview	PLINK	Haploview	PLINK	OR
			P-value	P-value	P-value	P-value	P-value	
2	*APIP*	APIP-1-13	0.023	0.023	0.146	0.003	0.002	0.70
	*ELF5*	**ELF5-1**	**0.005**	**0.004**	**0.005**	**3.27E-05**	**5.93E-05**	**0.54**
		**ELF5-3**	**0.018**	**0.016**	**0.037**	**0.325**	**0.241**	**0.63**
		**ELF5-5**	**0.002**	**0.002**	**9.23E-04**	**4.20E-07**	**1.44E-04**	**0.46**
		**ELF5-8**	**0.025**	**0.020**	**0.047**	**0.488**	**0.268**	**0.64**
	*CAT*	**CAT-E11-1**	**0.004**	**0.004**	**0.029**	**0.005**	**0.006**	**0.62**
		**CAT-5U-5**	**0.045**	**0.046**	**0.078**	**0.032**	**0.050**	**0.52**
		**CAT-5U-1**	**0.032**	**0.034**	**0.052**	**0.001**	**0.002**	**0.42**
	*HIPK3*	HIPK3-1	0.008	0.006	0.008	0.478	0.752	0.55
	*KIF18A*	KIF18A-E3-3	0.001	0.001	0.004	0.006	1.47E-03	0.58
	*LONP1*	LONP1-2	0.020	0.031	0.094	0.060	0.043	0.67
	*ADAMTS2*	ADAMTS2-E2021-1	0.034	0.034	0.019	4.00E-04	3.77E-05	1.38
	*COL23A1*	COL23A1-E2	0.008	0.008	0.003	3.22E-07	4.76E-10	1.60
12	*RAC3*	RAC3-I2-4	0.020	0.020	0.108	0.951	0.381	0.71
	*PYCR1*	PYCR1-1	5.40E-05	8.18E-05	1.74E-04	0.181	0.186	0.30
		PYCR1-E4-E5	8.80E-05	8.77E-05	7.96E-04	0.588	0.581	0.45
	*NPTX*	NPTX1-4-2	9.99E-06	1.49E-05	2.23E-05	0.053	0.021	0.94
		NPTX1-3	4.03E-04	8.00E-04	5.17E-05	1.90E-08	2.95E-09	1.52
	*CARD14*	CARD-1-1	0.059	0.035	0.015	0.394	0.419	1.34
		CARD-4-3	0.031	0.025	0.072	0.156	0.275	1.31
	*PRPSAP1*	PRP-I10	0.031	0.028	0.080	9.52E-04	8.91E-04	0.63
	*MIF4GD*	MIF-I7	0.014	0.014	0.008	0.490	0.304	3.00
	*ICT1*	ICT-I6-1	0.051	0.045	0.056	0.163	0.691	0.32

Note: SNPs in bold were in haplotypes significantly associated with scrotal hernia. Four SNPs in *ELF5* were found to constitute a haplotype together with the SNP EHF1 in *EHF*. Then after fine mapping, these four SNPs, together with three SNPs in CAT, formed another haplotype, more significantly associated with scrotal hernia (Figure 1E).

In order to further dissect the association from region II on SSC2, we added 26 markers surrounding the interesting genes, *ELF5* (1 MB region) and *KIF18A* (6 MB region) (Figure 1E, Table S1). At this stage of the genotyping and analyses, the SNP in intron 3 of *KIF18A* gave the highest association signal. Furthermore, we sequenced 15 exons (exons 2–16), except exon 1 of *KIF18A*, which were found in an unordered sequenced BAC clone CH242-13K2 on chromosome 2 (NCBI accession number: CU633215), and genotyped 14 SNPs from this gene. However, the newly genotyped SNPs from *KIF18A* were not significantly associated with scrotal hernia (p>0.05). In the catalase gene (*CAT*), which is very close to *ELF5* physically and oriented differently in human, three new SNPs, two in the 5′-untranslated region (UTR), CAT-5U-5 and CAT-5U-1, and one in intron 11, CAT-E11-1, were found to be associated significantly with scrotal hernia. The SNP CAT-E11-1 was still significant after multiple testing procedure (p<0.05). It seems that the closer the SNP to *ELF5*, the stronger the association (Table 1, Table S2). In addition, a haplotype (AAAAGCA) now composed of seven SNPs from *ELF5* and *CAT*, ELF5-1, ELF5-3, ELF5-5, ELF5-8, CAT-E11-1, CAT-5U-5 and CAT-5U-1, is highly significantly associated with scrotal hernia (p = 9.0E-04) (Figure 1E), which emphasizes that further sequencing could be directed upon this region.

**Figure 2:**
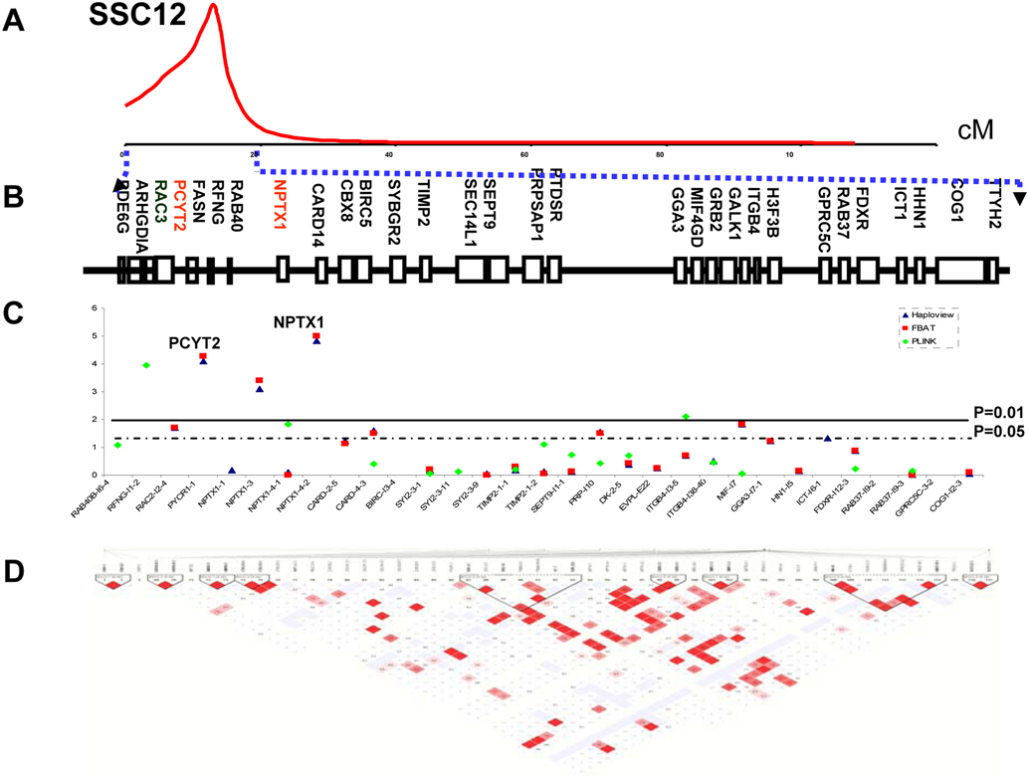
Extensive positional candidate gene analyses of the first 20 cM genomic region for hernia development on SSC12. A, The first 20 cM region was implied in hernia development on SSC12. B, Gene map was built based on the latest porcine physical FPC (fingerprint contigs) map (Sanger Porcine Genome Sequencing Project) (Table S1). C, Single marker association analyses. SNPs in *PYCR1* and *NPTX1* are highly significantly associated with scrotal hernia (global p<0.0001). D, LD heatmap constructed by Haploview shows that no extensive high LD block exists between these SNPs.

On SSC12, we focused on the first 20 cM region which supports a hernia QTL, and several genes in this region were found to be significantly associated with scrotal hernia (Figure 2, Table 1). Four SNPs in the pyrroline-5-carboxylate reductase 1 (*PYCR1*) and the neuronal pentraxin I (*NPTX1*), PYCR1-1, PYCR1-E4-E5, NPTX1-3 and NPTX1-4-2, were highly significantly associated with scrotal hernia (p<0.0001), respectively. The significance of the association signal is higher for these SNPs than those on SSC2. We observed low LD between them, as well for the remaining SNPs genotyped in this region.

We further analyzed the case-control dataset, composed of an admixture of several pig lines, two Duroc lines, two Landrace lines, one Large White line, and crossbred animals (Pietrain boars crossed to commercial females). This dataset gave the replication of the same significant association results as that in the family-based dataset, and seemed to demarcate the region associated with scrotal hernia (Table 1). On SSC2, two SNPs in *ELF5* (ELF5-1 and ELF5-5) were highly significantly associated with scrotal hernia (p<0.001). Two SNPs in *CAT* (CAT-E11-1 and CAT-5U-1) were significantly associated (p<0.01), and another SNP, CAT-5U-5, at p<0.05 level. The SNP in *HIPK3* was not associated any more. The same SNP in intron 3 of *KIF18A* (KIF18A-E3-3) was associated with scrotal hernia, like in the family-based analysis (p<0.01). The SNP in *COL23A1* (COL23A1-E2) was highly significantly associated with hernias (p<0.001) (Table S2). On SSC12, the SNP NPTX1-4-2 in *NPTX1* was found to be significantly associated with scrotal hernia in pigs (p<0.05), and another one, NPTX1-3, highly significant (p<0.001) also. However, SNPs from PYCR1 were not significant in case control samples.

## Discussion

After comprehensive SNP discovery and genotyping by using first a family-based analysis, which was replicated in another case-control dataset, we found that four regions surrounding *ELF5*, *KIF18A*, *COL23A1* on SSC2 and *NPTX1* on SSC12, respectively, may contain the genetic variants important for the development of the scrotal hernia in pigs.

The mapping of causal variants for complex disease traits requires the correctly assigned physical order and orientation of genomic sequences, and genetic relationship among genetic markers in specific genomic regions (LD status and functional annotation), which is still under development for pig genomics researchers now [23], [36].

Here, family-based analysis was first used to identify the four interesting regions, and then a case-control analysis similar to an admixture mapping method was used to replicate the results. Admixture mapping, or mapping by admixture linkage disequilibrium, is widely used in human disease studies now, to track the common underlying genetic factor(s) for different populations. It needs fewer markers, is more robust to allelic heterogeneity, and can achieve higher mapping resolution than traditional linkage studies [37]. We successfully replicated the results that revealed four genomic regions on SSC2 and SSC12 may underlie the development of porcine scrotal hernia. We observed some discrepancy of the significance level of the association analyses between the family-based and case-control datasets. The main reason for the discrepancy could be the experimental power, affected by the population structure, cryptic relatedness, sample size and so on [9], [21], [22]. The case-control design has more power to detect underlying factors than family-based design. However, it could also lead to more false positive results due to hidden population structures, unless the optimal selection of case and control samples took into consideration the genetic background, geographical distribution, age, and sex, etc. The family-based studies were more robust to population stratification, though with reduced power. Thus, many studies used several populations, family-based and/or case-control, to replicate and confirm the results.

Interestingly enough, genes fine mapped to be associated with scrotal hernia are implied in the pathway regulated by the estrogen receptors (*KIF18A* and *NPTX1*), collagen metabolism (*COL23A1*), and the epithelial-mesenchymal transition (EMT) (*ELF5*). These were three of the original five pathways that had been previously suggested. When treated with ICI 182,780 (the anti-estrogen fulvestrant), KIF18A, as a motor protein, was found to be able to bind to ERα, elicit the estrogen non-genomic response and activate the ERK-pathway as well [38], and during mitosis it can control the chromosome compression and movement [39]. The down-regulation of *NPTX1* was observed after ICI 182,780 treatment on rat efferent ductules, along with the altered expression of genes related to extracellular matrix organization, such as matrix metalloproteinase 7 (*MMP7*) [40]. These data suggest that estrogen can be related to the normal function of both KIF18A and NPTX1. Sex hormone deregulation has been considered as one of the reasons for hernia development [31]–[33], but significant association of genetic variants in *ESR1* and *ESR2* with scrotal hernia were not found using the same datasets [41]. We suspect that genetic polymorphisms in both *KIF18A* and *NPTX1*, as they could possibly be linked to estrogen, could also affect the function of estrogen, or be affected, and thus involved in the abnormal development of scrotal hernia, though the exact molecular mechanism is still unclear.

Aberrant collagen metabolism has been considered to be the main reason considered for hernia development [24]–[27], and *COL23A1* was identified to be associated with hernia development in our study. One of the major expression sites of *COL23A1* is skin [42], and its increased expression relates to the recurrence and metastasis of prostate cancer, which implies the existence of extracellular matrix turnover [43]. This could also suggest the involvement of *COL23A1* in hernia development. The *ELF5* gene, the exclusively epithelium-specific Ets transcription factor, can respond to hormonal cues, and determine the mammary gland development [44]–[46]. Its misexpression can also disrupt the specification and differentiation of epithelial cells [47], thus it is possibly involved in the process of the epithelial-mesenchymal transition [34], which is a remarkable characteristic of the etiology of scrotal hernia [1], [2], [4].

All these genes can potentially function in the development of hernia, but the exact molecular mechanism still needs further investigation. One common observation is that rates of hernias are less in purebred lines than in crossbreds, suggesting that the frequency of unfavorable alleles for one or more loci may be high in some lines and the opposite ones exist in the other lines. When crossed the frequency of undesirable alleles at many loci can come together. Given that this complex disease trait is under the influence of possibly many genetic and environmental factors, and the interaction between them, a specific molecular sub-network associated with the disease trait will need to be defined [17]. This, in turn, requires more time and energy to be put in the search for causal variants for scrotal hernia in pigs.

## Materials and Methods

### Ethics Statement

Animal care guidelines were followed according to the Institutional Animal Care and Use Committee (IACUC), Iowa State University.

### Animals

We collected 1,467 pigs born between 1991 and 2002, from 6 commercial pig breeding lines (Pietrain, Duroc, etc.) and pigs from crossbred herds, which were classified as affected or unaffected for scrotal hernias. These pigs can be divided into two different datasets with regard to with or without family information. The family-based Pietrain lines had 946 individuals from 298 pig nuclear families, including unaffected sires, dams, and male offspring (90% affected). The case-control dataset was composed of 100 unaffected and 421 affected males, which included animals derived from two Duroc lines, two Landrace lines, one Large White line, and crossbred animals (Pietrain boars crossed to commercial females). We extracted the genomic DNA of all animals, and adjusted the DNA concentration to 12.5 ng/µl.

### Discovery of Polymorphisms and Genotyping

We selected the positional and biological candidate genes after text mining the published data resources and literature. These genes were extensively examined for their comparative locations on pig chromosomes 2 and 12 (Table S1 and Figure S1). In addition, according to the comparative gene position on SSC2 to *Homo sapiens* (HSA) chromosomes 5, 11 and 19, and SSC12 to HSA17 (Figure S1), gene information (positions and functions) on four human chromosomes (5, 11, 17 and 19) were downloaded and curated (www.nature.com/nature/supplements/collections/humangenome/chromosomes/index.html). We further constructed the gene networks by putting them into PubGene (www.pubgene.org), not only to examine their relationships, but also to select more relevant candidate genes.

The latest information from the pig genome sequencing project from the Sanger Center (www.sanger.ac.uk/cgi-bin/humace/clone_status?speciesPig) was also checked and the sequences of whole BAC clones in the interesting regions were downloaded, and annotated by blasting against human genome sequence (Table S1). Furthermore, human mRNA transcript sequences of selected genes in the Ensembl database were used as templates to retrieve homologous pig sequences by a cross-species blast using the NCBI pig sequence database with HTGS or Trace-WGS options. PCR primers were then designed accordingly to amplify the available pig genomic sequences using Primer3 (http://frodo.wi.mit.edu/).

We optimized all the primers using a gradient PCR approach in a 10 µl system, and five individuals from each pig line (five in total) were used for single nucleotide polymorphism (SNP) discovery. After sequencing the PCR products, the sequences were aligned and compared using Sequencher software version 3.0 (Gene Codes, Ann Arbor, MI, USA). Some of the potential SNPs were confirmed by restriction fragment length polymorphism (PCR-RFLP) tests using suitable restriction endonucleases (New England Biolabs, Beverly, MA, USA).

The SNP genotyping was carried out commercially on the Sequenom MassARRAY® system. Two positive controls, two negative controls and one blank were put on the same plate for the purpose of quality control. Multiplex primers were designed using AssayDesign 3.1. After PCR amplification, samples were then spotted onto a 384 well SpectroChip® by a robot dispenser, which enabled an automated readout by laser excitation in a compact MALDI-TOF mass spectrometer. SNP genotypes were collected by the Typer 3.4 Software. Each SNP was checked to estimate the Hardy-Weinberg equilibrium status and the minor allele frequency (MAF).

### Statistical Analysis

All SNPs were analyzed individually by the transmission disequilibrium tests (TDT) on the family-trios and the case-control data. Three software packages widely used in human disease studies, FBAT (v2.0.2c), Haploview (v4.0), and PLINK (v1.02), were employed in this study [48]–[50]. Haploview was also used to examine the linkage disequilibrium status amongst all SNPs, by the two pairwise LD statistics, D' and the correlation coefficient (r^2^), as well as the haplotype association analyses.

Given that a large number of SNPs were genotyped, we evaluated the robustness of our results using the false discovery rate (FDR). FDR values (p<.20) were calculated using package ‘multtest’ in R (http://www.r-project.org) to evaluate the expected ratio of erroneous rejections of the null hypothesis to the total number of rejected hypotheses among all the genes or SNPs analyzed [51].

## Supporting Information

Figure S1 Positional candidate genes selected within four genomic regions associated with porcine scrotal hernia. Using the INRA comparative maps between Homo sapiens (HSA) and Sus scrofa, slight modification has been made for SSC2 and SSC12. On SSC2, three regions(Regions I–III) have been implied in pig hernia development. Markers defining these three regions were used to find genes HIPK3, CD59, CTSF and LONP1 (gene symbols in red), by BLAST using directly the marker sequence or BAC end sequence of those BACs containing the marker. Within the genomic region at the beginning of SSC12, 30 genes have been selected [S1–S8]. Only partial lists of the selected genes are shown here.
(0.12 MB DOC)

Figure S2 Gene network constructed for positional candidate genes. The gene networks were constructed by putting the selected candidate genes in the interesting genomic regions for porcine scrotal hernia into PubGene website (www.pubgene.org), not only to examine their relationships, but also to select out more relevant candidate genes in our target genomic regions.
(0.59 MB DOC)

Table S1 Positional Candidate Genes selected on SSC2 and SSC12.
(0.08 MB XLS)

Table S2 Association results for fine mapping regions linked to scrotal hernia in pigs (p-value).
(0.07 MB DOC)
