# Supplementary material for: Association and Haplotype Analyses of Positional Candidate Genes in Five Genomic Regions Linked to Scrotal Hernia in Commercial Pig Lines
Source: PLoS One. 2009 Mar 16;4(3):e4837. doi: 10.1371/journal.pone.0004837 (PMC2654076; doi:10.1371/journal.pone.0004837)
Supplement: Figure S1 — Positional candidate genes selected within four genomic regions associated with porcine scrotal hernia. Using the INRA comparative maps between Homo sapiens (HSA) and Sus scrofa, slight modification has been made for SSC2 and SSC12. On SSC2, three regions(Regions I–III) have been implied in pig hernia development. Markers defining these three regions were used to find genes HIPK3, CD59, CTSF and LONP1 (gene symbols in red), by BLAST using directly the marker sequence or BAC end sequence of those BACs containing the marker. Within the genomic region at the beginning of SSC12, 30 genes have been selected [S1–S8]. Only partial lists of the selected genes are shown here. (0.12 MB DOC) [file pone.0004837.s001.doc]

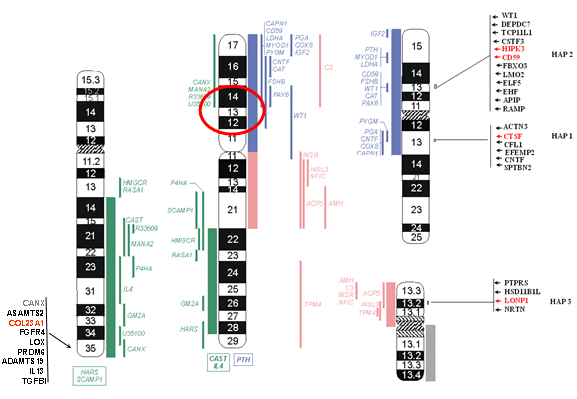


**SSC2**

**HSA11**

**HSA19**

**HSA5**


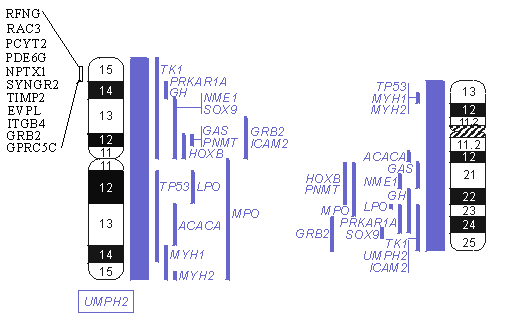


**SSC12**

**HSA17**

**Figure S1. Positional candidate genes selected within four genomic regions associated with porcine scrotal hernia**

Using the INRA comparative maps between *Homo sapiens* (HSA) and *Sus scrofa*, slight modification has been made for SSC2 and SSC12. On SSC2, three regions(Regions I-III) have been implied in pig hernia development. Markers defining these three regions were used to find genes *HIPK3*, *CD59*, *CTSF* and *LONP1* (gene symbols in red), by BLAST using directly the marker sequence or BAC end sequence of those BACs containing the marker. Within the genomic region at the beginning of SSC12, 30 genes have been selected [S1-S8]. Only partial lists of the selected genes are shown here.

**SUPPLEMENTARY REFERENCES**

S1. Jungerius, B.J. *et al*. *Anim Genet* **34**, 429-437 (2003).

S2. Lahbib-Mansais, Y. *et al*. *Genomics* **88**, 504-512 (2006).

S3. Liu, W.S. *et al*. *Genomics* **86**, 731-738 (2005).

S4. Liu, W.S. *et al*. *Cytogenet Genome Res* **120**, 157-163 (2008).

S5. Rink, A. *et al*. *Mamm Genome* **17**, 878-885 (2006).

S6. Rink, A. *et al*. *Mamm Genome* **13**, 578-587 (2002).

S7. Shimogiri, T. *et al*. *Anim Genet* **37**, 503-508 (2006a).

S8. Shimogiri, T. *et al*. *Cytogenet Genome Res* **112**, 114-120 (2006b).
