# Supplementary material for: Association and Haplotype Analyses of Positional Candidate Genes in Five Genomic Regions Linked to Scrotal Hernia in Commercial Pig Lines
Source: PLoS One. 2009 Mar 16;4(3):e4837. doi: 10.1371/journal.pone.0004837 (PMC2654076; doi:10.1371/journal.pone.0004837)
Supplement: Figure S2 — Gene network constructed for positional candidate genes. The gene networks were constructed by putting the selected candidate genes in the interesting genomic regions for porcine scrotal hernia into PubGene website (www.pubgene.org), not only to examine their relationships, but also to select out more relevant candidate genes in our target genomic regions. (0.59 MB DOC) [file pone.0004837.s002.doc]

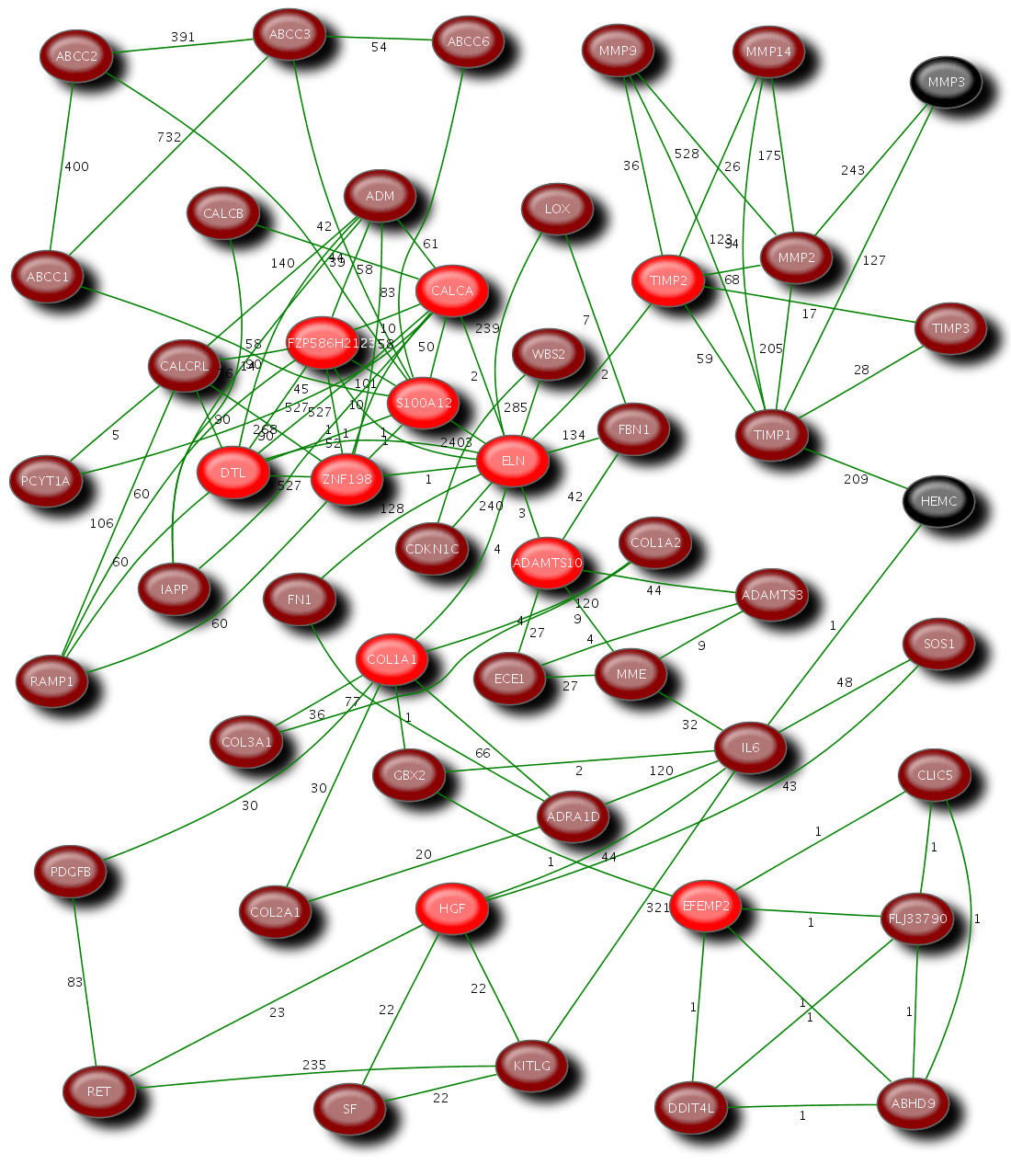


**Figure S2. Gene network constructed for positional candidate genes**

The gene networks were constructed by putting the selected candidate genes in the interesting genomic regions for porcine scrotal hernia into PubGene website ([www.pubgene.org](http://www.pubgene.org/)), not only to examine their relationships, but also to select out more relevant candidate genes in our target genomic regions.
