# Supplementary material for: Association and Haplotype Analyses of Positional Candidate Genes in Five Genomic Regions Linked to Scrotal Hernia in Commercial Pig Lines
Source: PLoS One. 2009 Mar 16;4(3):e4837. doi: 10.1371/journal.pone.0004837 (PMC2654076; doi:10.1371/journal.pone.0004837)
Supplement: Table S2 — Association results for fine mapping regions linked to scrotal hernia in pigs (p-value). (0.07 MB DOC) [file pone.0004837.s004.doc]

Table S2. Association results for fine mapping regions linked to scrotal hernia in pigs (p-value)

| SNP | Family | Case-Control |
| --- | --- | --- |
| **CD44-E2-1** | **0.8532** | **0.0238** |
| **CD44-E2-3** | **Na** | **0.1367** |
| **CD44-E15-1** | **0.5791** | **0.8281** |
| **CD44-E15-2** | **0.6115** | **0.0048** |
| APIP_1 | 1 | 0.589 |
| APIP_2 | 0.5835 | 4.00E-04 |
| APIP_3 | 0.6547 | 0.7375 |
| APIP_8 | 0.9489 | 8.41E-06 |
| APIP_4 | 1 | 0.001 |
| **APIP-1-13** | **0.0227** | **0.003** |
| **APIP_9** | **0.2741** | **0.0318** |
| **APIP-1-11** | **0.6988** | **0.0547** |
| **APIP-1-14** | **0.3519** | **0.0307** |
| EHF_2 | 0.0609 | 0.7893 |
| EHF_1 | 0.0776 | 0.0168 |
| ELF5_1 | 0.0037 | 3.27E-05 |
| ELF5_3 | 0.0158 | 0.325 |
| ELF5_5 | 0.002 | 4.20E-07 |
| ELF5_8 | 0.0196 | 0.488 |
| **CAT-E11-1** | **0.0042** | **0.0047** |
| **CAT-5U-5** | **0.0455** | **0.032** |
| **CAT-5U-1** | **0.0339** | **0.001** |
| **M11S1-E1-1** | **1** | **2.56E-12** |
| FBXO3_12 | 0.8059 | 4.00E-04 |
| FBXO3_2 | 0.1724 | 0.5642 |
| FBXO3_7 | 0.0701 | 0.0148 |
| HIPK3_1 | 0.0057 | 0.478 |
| TCP11L1-3UTR | 0.0896 | 0.0919 |
| **DEPDC7** | **0.718** | **0.6159** |
| WT1_1 | 0.593 | 2.14E-21 |
| PAX6-E13-2 | 0.2132 | 1.05E-06 |
| **DCDC1-E19-1** | **0.5952** | **0.0029** |
| **DCDC1-E19-4** | **0.6362** | **0.0031** |
| **FLJ38968-E4-1** | **0.343** | **0.024** |
| METT5D1-E2-1 | 0.4581 | 0.0426 |
| **KIF18A-I1-1-3** | **0.5271** | **0.0478** |
| **KIF18A-E2-1** | **0.2575** | **0.0045** |
| KIF18A-E3-3 | 0.0014 | 6.00E-03 |
| **KIF18A-I3-2-1** | **0.4631** | **0.0271** |
| **KIF18A-I3-3-1** | **0.3991** | **0.0454** |
| **KIF18A-I3-3-4** | **0.5961** | **0.0342** |
| **KIF18A-I3-4-1** | **0.4631** | **0.0405** |
| **KIF18A-I3-4-2** | **0.6698** | **0.0818** |
| **KIF18A-E6-1** | **0.5271** | **0.0161** |
| **KIF18A-E17-2** | **0.6056** | **9.00E-04** |
| **ENST00000314102-E1-1** | **0.1398** | **3.00E-04** |

Note: 26 SNPs for fine mapping are in order of their physical positions, and are in bold. Na, not available.
